# Report on the 9th EONS Congress, Istanbul, Turkey, 18–19 September 2014: nursing highlights

**DOI:** 10.3332/ecancer.2014.481

**Published:** 2014-11-13

**Authors:** D Lichosik, R Caruso

**Affiliations:** 1 European Institute of Oncology/IEO Education, Via Ripamonti 435, Milan 20141, Italy; 2 Nursing Degree Course, University of Milan, IRCCS Policlinico, San Donato 20097, Italy

**Keywords:** 9th EONS Congress, conference highlights, oncology nursing

## Abstract

The European Oncology Nursing Society (EONS), in partnership with the Oncology Nursing Association of Turkey (TONA), organised the 9th EONS Congress in Istanbul, Turkey, 18–19 September 2014. The Congress venue was in Harbiye Cultural Centre and Istanbul Military Museum and offered two days of unprecedented scientific, educational, and networking opportunities for all stakeholders active in the field of oncology nursing around the world.

EONS is a pan-European organisation dedicated to the support and development of cancer nurses. Through individual members and national societies EONS engages in projects to help nurses develop their skills, network with each other, and raise the profile of cancer nursing across Europe.

This biennial Congress brings together cancer nurses across the globe from many fields of expertise to ‘Celebrate Excellence in Cancer Nursing’ through interactive sessions, lectures, and collegial networking. The format was specifically designed to meet the needs of nurses working in the field of cancer care, education, and research.

## Introduction

The European Oncology Nursing Society (EONS) was founded in 1984 as ‘the Fellowship of European Oncology Nursing Societies’. The founding chairperson was Mrs Rosette Poletti (Italian).

The EONS is an independent, not-for-profit, voluntary organisation which was established 30 years ago and is now celebrating its anniversary. EONS has developed as a professional organisation, a voice for cancer nursing in Europe, and an important partner in multi-organisational efforts to improve cancer care and patient outcomes. The EONS is working to develop cancer nursing as a specialism in all European countries and also to improve collaboration between countries to improve nursing roles in cancer care. The strength of EONS comes from its cooperation with a range of multidisciplinary organisations with which it collaborates to optimise the nursing contribution to cancer care in Europe. The Society recognises the diverse aspects of nursing across Europe and strives to work towards equitable basic training and continuing education for nurses in the area of cancer nursing. The activities of EONS are directed towards empowering cancer nurses to attain due recognition as specialists.

The EONS originally had its headquarters at the Royal Marsden Hospital in London. The EONS Secretariat was relocated to Brussels in 1997. An Executive Board, Advisory Council, and the General Membership make up the organisational structure of EONS. These three bodies collaborate to identify the goals and strategies of the Society and to implement activities which support the mission of EONS. The Membership governs the Society through activities including voting on constitutional changes, discussion of strategic issues, and nomination and election of members of the Executive Board.

**EONS vision:** That all people affected by cancer across Europe will benefit from the care of well-educated, well-informed, and highly competent cancer nurses, who will play a central role in providing support, promoting health, and improving clinical outcomes.

**EONS mission:** Working in partnership to develop and promote excellence in cancer nursing practice through education, research, leadership, and support to cancer nurses across Europe.

**EONS aims:** Promote and develop the practice of cancer nursing in European countries, thus improving the quality of care that cancer patients receive; develop educational resources for nurses engaged in caring for patients with cancer, promote educational programmes, and identify and build on a body of knowledge specific to cancer nursing; encourage development of and participation in collaborative research and the publication of results; encourage exchange programmes; provide a means of communication between oncology nursing groups and individuals engaged in cancer nursing.

## The strategic CARE plan

The EONS board reviewed and updated the strategy during the EONS board meeting in February 2013. The review focused on assessing the changing educational needs of cancer nurses and the changing environment in which they work. The EONS board agreed that the existing CARE strategy remained valid and addressed members’ needs.

**The CARE strategy is based on**

- Communication
- Advocacy
- Research
- Education.

The EONS 9th Congress took place in 2014, its 30th anniversary year. Oncology nurses from all over Europe (over 450 nurses) headed to Istanbul to ‘Celebrate Excellence in Cancer Nursing’ for over two days of learning, discussion, and networking. The EONS-9 was run in conjunction with the annual congress of TONA, the Turkish Oncology Nursing Society. This biennial Congress brings together cancer nurses across the globe from many fields of expertise to ‘Celebrate Excellence in Cancer Nursing’ through interactive sessions, lectures, and collegial networking. The format was specifically designed to meet the needs of nurses working in the field of cancer care, education, and research. This together with a first class panel of speakers presenting on the latest trends and developments within their specialty consequently drew in an audience comprising all stakeholders in the field of oncology nursing. Hot topics included sessions such as: ‘Reintegration and rehabilitation after cancer treatment’, ‘Lifestyle and cancer’, ‘Leadership’, and ‘Patient safety’, which took place across two days. Other sessions focused on palliative care, chemo-induced neutropenia, complexity of care, management of side effects, polypharmacy, rare cancers, ambulatory care, body image changes, new oncology research, and tumour specific topics, plus parallel sessions, workshops, and satellite symposia.

**Proffered papers:** A total of 79 proffered papers were presented during the main and parallel sessions, from European, American, and Asian countries. The major issues which were highlighted include:

- **Psychosocial issues and support**
  - Lifestyle and cancer
  - Lifestyle and survivorship
  - Prostate cancer, European Association of Uology Nurses/European Oncology Nursing Society (EAUN/EONS)
  - Follow-up and psychosocial needs of young adult cancer survivors
- **Cancer Nursing Developments**
  - Leadership International Society of Nurses in Cancer Care/European Oncology Nursing Society (ISNCC/EONS)
  - Complementary and alternative medicine (CAM)
  - Body image changes
  - Colorectal cancer (Europacolon/EONS)

**Poster session:** A total of 185 posters were presented during the poster session. The major issues which were highlighted include:

- Advocacy planning and policy
- Educations for patients, relatives, and nurses
- Lifestyle and cancer
- Management of cancer services
- News developments
- Oncology nursing research
- Palliative care
- Patient advocacy and support
- Patient safety
- Psychosocial issues and support
- Sexuality issues
- Survivorship and late effects
- Symptom management
- The economics of cancer and technology.

## Poster session

T. Ozkan from Erciyes University, Kayseri, Turkey presented a poster on ‘Sexuality and cancer’, which reported the importance of information given to patients and relatives about coping with cancer and its impact on the patient’s body, specific diseases, treatments methods, and changes in the relevant parts of the body. Health professionals should not ignore such an important issue which will directly affect the quality of life of an individual, and they should fulfill their responsibilities in this area.

R. Caruso and others from the Nursing Degree Course, University of Milan, IRCCS Policlinico San Donato, Italy and European Institute of Oncology, Milan, Italy presented a poster titled ‘Cancer patients experiences of enhanced recovery after general surgery’, in a poster session on Oncology Nursing Research.

The presented study aimed to increase the understanding of how cancer patients view, cope with, and experience general surgery enhanced recovery programmes. Patient’s discharge is an event that often leads to uncertainty and a sense of loss, and the time of hospitalisation is considerably reduced, especially for surgical treatments. The benefits of reducing the time of hospitalisations are numerous and well-studied. This strategy is also used by many hospitals to improve clinical outcomes and to reduce the costs of hospitalisations [3] and is known as enhanced recovery after surgery (ERAS). This study was a qualitative research analysis, using semi-structured interviews to investigate issues of significance to cancer patient’s experiences of enhanced recovery after general surgery. The end result is a report containing five major themes which summarise the main problems felt by patients: happiness at the idea of being able to return home; uncertainty through lack of preparation for discharge; uncertainty through lack of information; uncertainty about ‘being at home’; uncertainty about the future. It is evident that the categories identified encompass both the clinical and organisational aspects of nursing practice. Aspects such as the uncertainty of ‘being at home’ were deeply felt by the participants.

This qualitative study is full of spontaneous and unexpected elements. These interviews have produced high-quality data, suggestions, and proposals, which can and should be used to try to identify as adequately as possible the needs of our hospitalised patients before their discharge.

Hiroko Komatsu and Kaori Yagoasaki from the Faculty of Nursing and Medical Care, Kelo University of Japan, presented an interesting poster: ‘Power of Nursing: What Makes Nursing Unique?’ Using a grounded theory approach, they conducted a qualitative study with four focus group interviews of 21 oncology nurses currently providing counseling and support services for all types of cancer patients in Japan. The objective of this qualitative study was to understand the experiences of oncology nurses in patient counseling and support services in the ambulatory care setting, and to explore the uniqueness of nursing.

The conceptual model of the power of nursing was developed from the results of the present study to guide patients across the cancer trajectory which emerged as three phases:

1. Connecting with a patient (shared needs)—Oncology nurses guide patients by identifying a patient’s true needs based on an established relationship and giving special attention to the patient as an individual.
2. Personalised coordination (shared action)—The nurses focus on the patient’s daily life and provide personalised coordination.
3. Realising the patient’s potential (reassurance)—The nurses develop the patient’s potential. Patient-centred care can be provided in non-physical care settings such as a counseling and support service.

This conceptual model describes the uniqueness and significance of nursing. It can be used as a tool to realise the full potential of nurses in practice, and to build the professional identity of nurses. Oncology nurses can take a leadership role in enhancing the visibility of nurses in multidisciplinary environments.

The year 2014 is the first year in which the EONS Congress had an exclusive focus on education. This meant a wide choice of workshops and opportunities to learn about the latest research in the field-as well as the usual opportunities to network with colleagues from other countries.

## Workshops

‘Integrating Screening for Distress in Nursing Practice’-IPOS/ISNCC/EONS, a workshop coordinated by G. Cummings from Canada, with the participation of D. McLeod and A. Krol from the Netherlands, provided the possibility to learn about new clinical practice guidelines (Distress Thermometer) in relation to the screening of patients’ physical, psychological, social, and emotional well-being.

‘Managing Sexual Consequences of Cancer and its Treatments’, a workshop coordinated by I. White from the UK had the following objectives:

- Identify the aetiology and prevalence of common treatment-induced sexual difficulties encountered in cancer care.
- Explore the management of desire, arousal, orgasmic and sexual pain disorders for individuals/couples affected by cancer
- Outline levels of service provision for individuals/couples with treatment-induced sexual difficulties.

Promoting sexual recovery after cancer is an important duty for all professionals involved in cancer treatment.

## Educational session

‘Multi-Professional Management of Cancer-Related Pain’ coordinated by A. Margulies from Switzerland and B. Quinn from the UK.

Topics in this session reminded us that pain is the most common symptom experienced by patients with cancer and for many patients it is the most feared symptom. W.H. Oldenmenger from the Erasmus MC Cancer Institute, Rotterdam, The Netherlands, in his presentation ‘Optimal pain control-why is it so hard to get it right?’ reported that cancer-related pain is poorly managed. Although oncology professionals make numerous diagnostic and therapeutic decisions about pain in everyday clinical practice, they frequently make poor clinical decisions, e.g., underestimating the severity of the patient’s pain, giving too small a dose of an analgesic over a longer interval than necessary, or overestimating the effectiveness of an intervention. However, there are also many other factors, including inadequate collection and interpretation of data, patient’s reluctance and inability to report pain, poor multi-professional collaboration, and myths and misconceptions surrounding opioid use. Patients and their relatives have many barriers and misconceptions related to pain, analgesics, and side-effects which could influence their pain treatment.

## Parallel session

### ‘Lifestyle and cancer’—coordinated by B. Grube from Denmark

A. Anderson from the University of Dundee, School of Nursing and Midwifery, UK, spoke about ‘Healthy diet and lifestyle for cancer patients’, reporting that the American Cancer Society and the World Cancer Research Fund highlight the importance of achieving and maintaining an optimum body weight, engaging in physical activity (whilst avoiding inactivity), and following a dietary pattern that is rich in fruits, vegetables, and whole grains with low intakes of alcohol, tobacco, and red and processed meat. For cancer patients, healthy ways of life offer an opportunity to improve treatment outcomes, quality of life, psychological and physical condition, and well-being. Sensitive, well-informed practical strategies have been demonstrated to initiate behavioural changes in cancer patients but evidence based approaches and protocols for good practice have not yet been widely disseminated and implemented. Ensuring that treatment plans include personalised lifestyle dimensions offer patients the opportunities to maximise health gains beyond drug therapies.

### ‘Breast cancer’—coordinated by P. Fernandez-Ortega from Spain

M. Nilsson and others from the Karolinska Institute, Stockholm, Sweden presented an interesting study titled ‘Return to work after breast cancer: women’s experiences of encounters with different stakeholders’. Breast cancer is the most common cancer in women worldwide. Half of the women diagnosed in Europe are of working age, and most survive; thus, knowledge is needed on how to provide support regarding work-related issues, to prevent exclusion from the labor market. Questionnaire data, register, and focus group data have been collected and analysed. Data were analysed by quantitative content analysis. The Karolinska Institute researchers stressed that it is very important to consider how the information, encounters with stakeholders and planning of care can be optimised in order to facilitate return to work after breast cancer diagnosis and treatment.

### ‘Complexity of care in rare cancers’—coordinated by D. Kelly from the UK

G. Gatta and others from the National Cancer Institute, Epidemiology Unit, Milan, Italy, presented a study on Rare Cancers and spoke about the project Surveillance of rare cancers in Europe (RARECARE). According to RARECARE definition (incidence <6/100,000/years), around four million people in the European Union (EU) are affected by rare cancers. Researchers’ estimates of the rare cancer burden in Europe provide a first indication of the size of the public health problem due to these diseases and constitute a useful base for further research. Centres of excellence for rare cancers could provide the necessary organisational structure and critical mass for carrying out clinical trials ad developing alternative approaches to clinical experimentation, declared researchers.

EONS-9 included a session titled ‘New Oncology Nursing Research’, which was an opportunity for two nurse researchers to present their work for the first time. Both researchers were recipients of the EONS Novice Research Dissemination Award, which was initiated by the EONS Research Working Group to encourage cancer nurses who have previously not presented at an international conference, to disseminate their research:

Joyce Roijen of The Netherlands presented her work on ‘Fertility counseling in young women with breast cancer before adjuvant chemotherapy’.

Elif Sözery of Turkey presented the results from her study on ‘Resilience and burnout among oncology nurses’.

The European School of Oncology (ESO) has pioneered the concept of the e-grand round, a live online teaching session. ESO offers these sessions free of charge every week, giving health professionals the opportunity to learn from a specialist in their field and ask them questions directly. Recordings of the sessions are available for up to six months afterwards and also provide an important educational resource. Many of these will be of relevance to nursing practice. New feature: all participants in e-grand rounds will now be able to send questions to the experts before, during and after the live sessions.

## The masterclass in oncology nursing

Anita Margulies from Switzerland and Lena Sharp from Sweden presented the programme of the Masterclass Course in Oncology Nursing for 2015.

### 7–12 March 2015 Ermatingen (Lake Constance), Switzerland

**Co-chairs: Anita Margulies, Switzerland and Lena Sharp, Sweden**

The Masterclass in oncology programme is designed for advanced oncology nurses as a multi-professional joint event. Five intensive days of full immersion in up-to-date oncology creates a collective spirit of teaching and learning to improving clinical skills and patient care.

In joint clinical sessions, nurses, together with physicians, update their clinical knowledge about the latest issues for the management of breast, gynecological, prostate, colorectal cancer, and lung cancer, with an international faculty of experts. Special joint sessions in supportive care, communication skills and palliative care are supported by workshops and coaching to explore nursing practice in these speciality areas.

Specific nursing-oriented sessions explore specialist practice skills such as advanced assessment, decision making, communication skills, complex symptom management, care of the older patient, psychosexual consequences of cancer therapy, long-term adverse effects of cancer therapy, and managing advanced disease.

Practical training is offered in smaller group optional sessions and coaching groups focusing on skills required for the management of active therapy and follow-up, for example, assessing psychological distress, managing sexual concerns raised by patients. Furthermore reviewing and understanding the growing evidence base for nursing interventions plus reflections on the participant’s role in clinical leadership.

## Cancer nursing partnership (CNP)

The Cancer nursing partnership (CNP) is a partnership of key nursing organisations and communities of practice who are working collaboratively to implement improvements in cancer care. The CNP is currently working to support the delivery of the recovery package to people living with and beyond cancer across the UK. The recovery package is a series of key interventions which, when delivered together, can greatly improve outcomes for people living with and beyond cancer and reduce unnecessary hospital attendance. These consist of:

- A holistic assessment of the person’s needs, and a tailored plan for meeting these, to enable them to maximise their well being posttreatment
- A treatment summary that explains to the general practitioner (GP) and the individual what treatment has taken place and what should happen next, giving them the confidence to self-manage their condition
- A cancer care review with their GP within six months, to ensure they are getting the support they need
- A health and well being event which aims to educate and empower the person to manage their condition and keep themselves as fit and healthy as possible.

As well as these fundamentals, other areas that make a big difference are:

- Rehabilitation, physical activity, and self-management classes to help people manage their condition and keep themselves as well as possible
- Back to work support helps people move back into employment where appropriate, so that they can be financially self-sufficient, and can benefit from the well being gains of being in work
- Financial advice helps people to get out of, and avoid falling into, financial difficulties which can lead to stress, mental health problems, and other well being impacts.

## EONS9 satellite symposia overview

Bayer satellite symposium. Thursday 18 September, 16:00–17:30, Inonu Hall - ‘Metastatic castration-resistant prostate cancer (CRPC) patients with bone metastases’

A panel of experts addressed the challenges of managing patients with CRPC and bone metastases, the role of radium-223, and discussed best practices for optimising patient care.

Oncology nurses have a critical role in detecting symptoms of cancer progression and in connecting the multidisciplinary team of urologists, oncologists, and nuclear medicine specialists to address the needs of patients with (CRPC).

## Conclusion

The plenary lecture reminded the audience of the history of EONS, the development of education for oncology nurses, research activity in the field of oncology, improvement of services for oncology patients, and indicated new challenges.

The strategic workshop ended with comments about patient active involvement in the decision-making process and therapy process.

The development of new services needed for long term treated patients, palliative care, and survivors was discussed.

During the EONS 9 Congress, nurses had the opportunity to receive information and complimentary material about the Society and scientific events, visit the EONS booth and contact EONS board members.

It was an exciting event in the lively city of Istanbul.

The next Congress, EONS-10, will be held in Ireland in 2016.

## Figures and Tables

**Figure 1.**
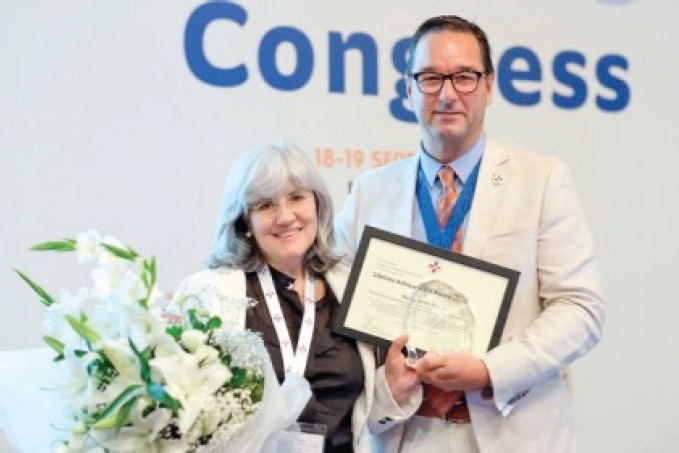
Paz Fernandez Ortega receiving her award.
